# Determinants, Pathways, and Outcomes of Health Literacy in India: A System Dynamics Modelling Study

**DOI:** 10.7759/cureus.99661

**Published:** 2025-12-19

**Authors:** Arjun B, Fathima Hassan, Akhil Jaison

**Affiliations:** 1 Community and Family Medicine, All India Institute of Medical Sciences, Bhopal, Bhopal, IND

**Keywords:** determinants of health, health literacy, health systems, systems dynamic modelling, systems thinking

## Abstract

Health literacy is a critical determinant of health. While many studies identify linear associations, the dynamic feedback mechanisms that create policy resistance and perpetuate health inequities remain poorly understood and studied. This study aims to develop a conceptual model of health literacy contextual for India, grounded in a systematic review of the literature, to identify key feedback loops and to test the comparative effectiveness of different policy interventions via system dynamics modelling. We conducted a systematic review to identify determinants, pathways and outcomes of health literacy in India. The search yielded 559 records. Two independent reviewers did title and abstract screening, resulting in 39 articles selected for full-text review. After four records could not be retrieved, 35 full texts were assessed for inclusion in the study, and 30 studies met the final inclusion criteria. Data extraction was done, and a Causal Loop Diagram (CLD) was developed iteratively. This conceptual model was then quantified into a Stock-and-Flow (S&F) simulation model using Python. We conducted a sensitivity analysis and simulated five distinct policy scenarios over 30 years. The model's behaviour is dominated by two reinforcing vicious feedback loops: 1) the 'Health Disparity Trap' (R1), where low socioeconomic status drives low health literacy and poor health, which in turn reinforces poverty; and 2) the 'System Trust Spiral' (R2), where poor health outcomes erode public trust, leading to care avoidance of care and further health decline of health. After running the simulation, the total population with poor health outcomes was 55.6 million, with 95% CI 43.8M-65.5M in the baseline scenario. Isolated interventions failed to show a significant improvement in the total population with poor health outcomes from the baseline. A Stacked Intervention, which was multimodal, produced the strongest effect, reducing the mean population with poor health outcomes to 35.7 million (95% CI: 24.9M-47.6 M). Health literacy is a complex and adaptive construct influenced by reinforcing mechanisms that make it resistant to single-point interventions. Policy must move from isolated projects to a multi-pronged, systemic strategy to produce significant impact.

## Introduction and background

Health literacy is increasingly recognised as a foundational public health asset. It represents the capacity of individuals and communities to access, understand, appraise, and apply health information. This is crucial for making sound decisions regarding healthcare, disease prevention, and health promotion [1]. In India, with its vast demographic, linguistic, and social diversity, health literacy acts as a critical mediator that could alter the course of a wide range of public health challenges, ranging from the growing burden of non-communicable diseases to improving maternal and child health outcomes [2-4].

Previous Indian literature has successfully identified numerous linear associations, linking low health literacy to upstream determinants like education, socioeconomic status, and gender [5,6], and to downstream outcomes like poor medication adherence [7], uncontrolled hypertension [2], and child stunting [4]. However, these factors do not exist in isolation. The interplay between poverty, education, systemic mistrust, and the healthcare system itself creates a complex web of causality. These factors often interact and reinforce one another, creating "feedback loops" that trap populations in cycles of poor health outcomes and poverty. Low health literacy can lead to poor outcomes, which may foster mistrust in the health system. This mistrust, in turn, can lead to care avoidance, which further worsens health outcomes, creating a vicious cycle [8].

While many conceptual frameworks for health literacy exist, most remain qualitative in nature. To date, few studies have attempted to quantify these interactions and feedback loops in the Indian context. This gap leaves policymakers without the tools to understand why well-intentioned, isolated interventions may fail, or to identify which combination of interventions might be most effective [9]. Quantitative models enable the simulation of policies over time, allowing the testing of hypotheses about systemic behaviour and the identification of high-yielding interventions before committing resources in the real world. This study aims to address this gap by developing a conceptual model of health literacy contextual for India, grounded in a systematic review of the literature, to identify key feedback loops and to test the comparative effectiveness of different policy interventions via system dynamics modelling [10].

## Review

Methods

This study was conducted in two phases: (1) a systematic review to identify determinants, pathways and outcomes to build a conceptual model of health literacy and gather parameters for subsequently building a Stock-and-Flow model, and (2) to simulate the impact of various policy scenarios.

Eligibility Criteria

This review included primary research studies, quantitative, qualitative, or mixed-methods studies, and editorials that discuss the determinants, pathways, and outcomes of health literacy and examine their linear relationships. To be included, studies had to be relevant to the Indian context and discuss the determinants, pathways, and outcomes of health literacy, as well as their relationships. Review articles were excluded from the analysis.

Search Strategy

A systematic search was conducted in PubMed and Google Scholar for articles published up to October 1, 2025. The review was conducted in accordance with the Preferred Reporting Items for Systematic Reviews and Meta-Analyses (PRISMA) guidelines [11]. The search strategy combined Medical Subject Headings (MeSH) and keywords relevant to the concept of health literacy, including terms such as "functional," "communicative," and "critical health literacy", and the location of the study, including names of specific states (Appendix 1).

**Figure 1:**
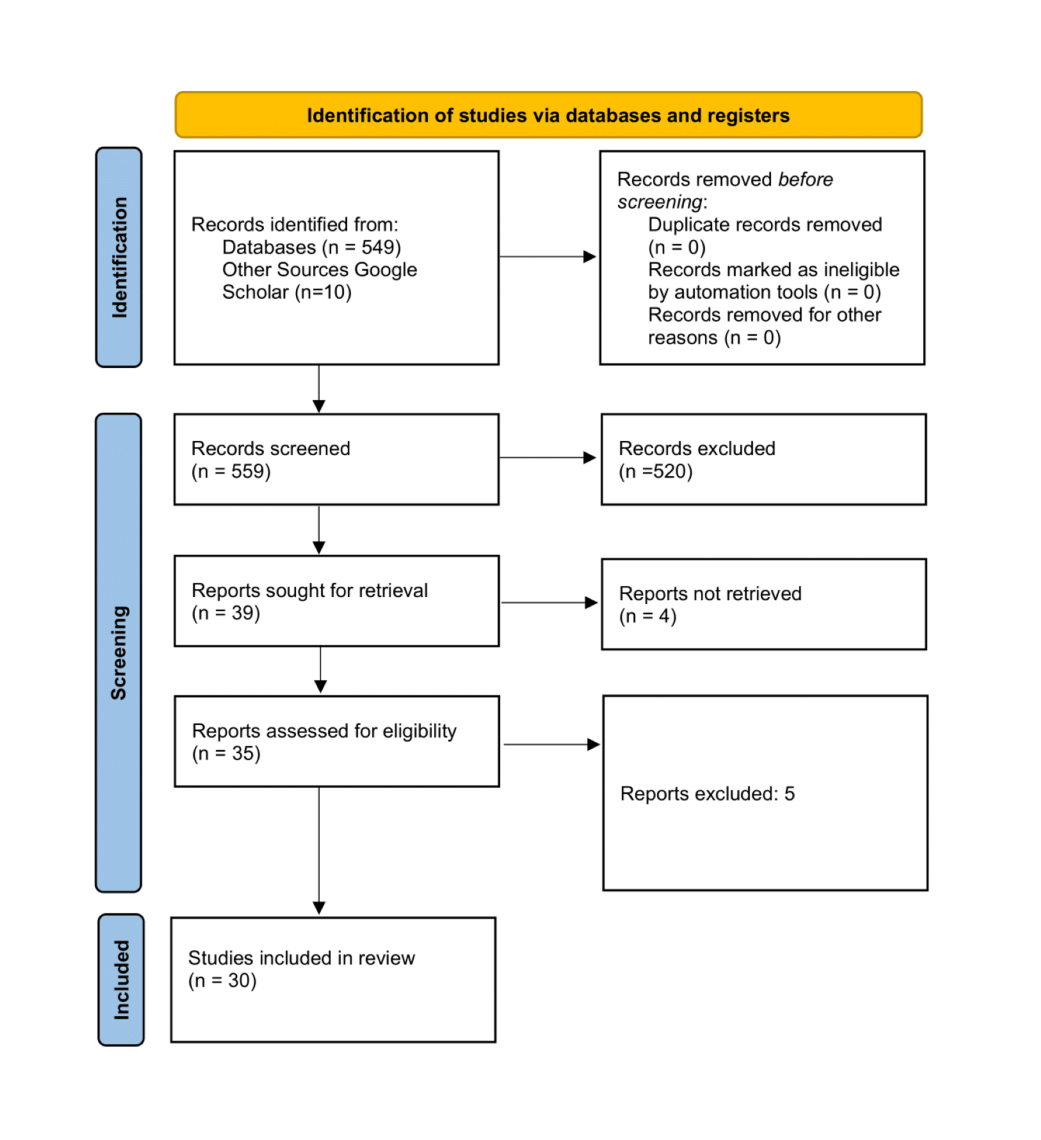
Preferred Reporting Items for Systematic Reviews and Meta-Analyses (PRISMA) flow diagram

The search yielded 549 records from the PubMed database and 10 from Google Scholar. After removing duplicates, two independent reviewers screened titles and abstracts. This resulted in 39 articles selected for full-text review. We were unable to retrieve four of these records. Of the 35 full-text articles assessed for eligibility, 30 studies met the final inclusion criteria and were included in the review (Figure 1). Data from the 30 included studies were extracted onto a data extraction sheet. Key variables extracted included study design, setting, population, Health literacy measurement tool, and key findings, with a specific focus on quantitative effect measures and qualitative themes related to determinants and pathways. The quality appraisal of the included studies was conducted using the Mixed Methods Appraisal Tool (MMAT).

Model Building

The extracted data was first synthesised into a qualitative Causal Loop Diagram (CLD) to map the system's structure. This process identified the core components (Determinants, Pathways, Outcomes) and three key feedback loops that govern the system's behaviour, a vicious cycle of disparity (Reinforcing Loop 1, or R1), a vicious cycle of system mistrust (Reinforcing Loop 2, or R2), and a virtuous cycle of empowerment (Balancing Loop 1, or B1). The CLD was then quantified into a Stock-and-Flow simulation model using Python (v3.10) [12] with NumPy and Matplotlib libraries [13,14]. The model can be understood as a system of four interconnected stocks representing key population segments and system states. The simulation tracks how the distribution of 100 million people across these stocks changes over a 30-year period (Appendix 2).

Population with low health literacy: this stock is depleted by the health literacy improvement flow, which represents individuals gaining health literacy and moving to the population with high health literacy stock. Functionally, this stock represents the at-risk population. The model applies the effect of low health literacy on health [2], causing this population to transition to a state of poor health at an accelerated rate.

Population with high health literacy: health literacy improvement flow from the low health literacy population fills it. Functionally, this is the "protected" population. The model applies a protective effect of high health literacy on health [4], causing individuals in this stock to enter a state of poor health at a slower rate.

Population with poor health outcomes: This stock is our primary outcome indicator. It is filled from both the low health literacy and high health literacy stocks and is depleted by the recovery outflow.

Public trust: This stock is measured on a 0-100 scale. It is filled by a trust-building flow and depleted by a trust erosion flow. If the population with poor health outcomes stock exceeds a predefined threshold, the trust erosion flow is activated, and public trust declines. Functionally, this stock creates "policy resistance," as a decline in trust renders empowerment interventions less effective (Appendices 3, 4).

Policy Scenarios

We simulated five policy scenarios, with all interventions activated at Year 5 of the 30-year run. Scenario A (Baseline), a "do nothing" scenario, represents the system's natural trajectory. Scenario B (Empowerment) tests an IEC health literacy intervention [15-17]. Scenario C (Disparity Mitigation) tests a hypothesised social support policy aimed directly at the health outcome. Scenario D (Trust-Building) tests a policy focused on improving healthcare quality and system trust. A hypothesised intervention, such as provider communication training. Scenario E (Stacked Interventions) simulates a multi-pronged, systemic strategy by activating Scenarios B, C, and D simultaneously (Appendix 5).

We conducted a 100-run bootstrap sensitivity analysis. In each run, data-driven parameters were sampled from normal distributions defined by their 95% CIs and assumed parameters were varied by ±20%. We report the mean results for all scenarios and the 95% CIs for Scenarios A and E.

Results

Systematic Review and Model Structure

**Figure 2:**
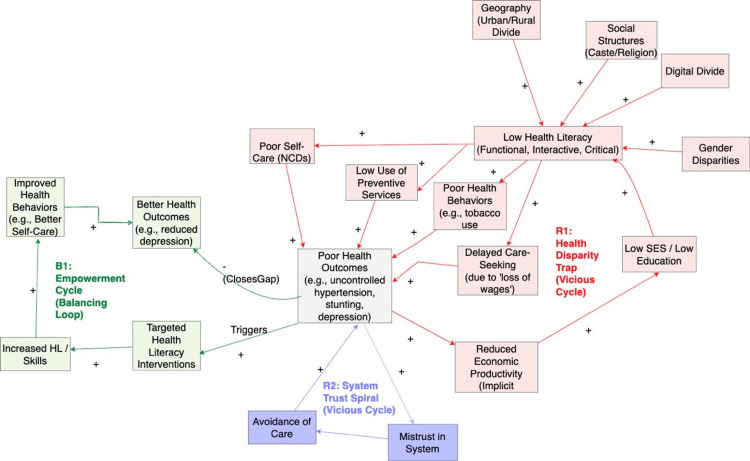
Causal Loop Diagram of Health Literacy NCDs: non-communicable diseases; SES: socioeconomic status

A summary of the included studies is provided in Table 1. A narrative synthesis approach was used to develop the conceptual model. Data on determinants, pathways, and outcomes were synthesized thematically. Causal statements and reported effect sizes were then mapped, translating the synthesised narrative into a Causal Loop Diagram visualised in Figure 2.

**Table 1:** Summary of Studies Included in the Systematic Review HLS-EU-Q16: European Health Literacy Survey Questionnaire, 16-item; NCD: non-communicable disease

First Author, Year	Study Design	Target Population	Key Findings
Ahmad et al., 2021 [17]	Cross-sectional	Women from rural Uttar Pradesh	An integrated microfinance and health literacy program improved knowledge of maternal danger signs.
Ahmad et al., 2022 [15]	Quasi-experimental	Pregnant/recently delivered women from rural Uttar Pradesh	An integrated microfinance and health literacy program improved birth preparedness and complication readiness.
Baliga, 2019 [18]	Editorial	Children and parents	Proposes comprehensive solutions focusing on education, policy, and service delivery to improve child oral health literacy.
Chauhan & Trivedi, 2024 [3]	Mixed-method	Hypertensive adults from Gujarat	Illiteracy about tobacco hazards was significantly associated with tobacco addiction among hypertensive patients.
Das, 2020 [19]	Cross-sectional	Adult population from Ghaziabad, Uttar Pradesh	50% had inadequate oral health literacy, which was significantly associated with lower socioeconomic status and poorer oral health status.
Douglass et al., 2021 [20]	Mixed-methods	Emergency Department clinicians	Low health literacy among patients was a major barrier to effective communication in multilingual Indian Emergency Departments.
Dsouza et al., 2021 [21]	Cross-sectional	Hindi and Kannada-speaking individuals	Validated the HLS-EU-Q16 for use in India.
Gautam et al., 2021 [16]	Cross-sectional	Chronic disease patients from Rajasthan	65.8% had insufficient health literacy, which was a significant predictor of lower COVID-19 awareness and preventive behaviour.
Gokdemir et al., 2024 [22]	Editorial	General	Discusses micro, meso, and macro-level factors influencing health literacy and highlights the need for culturally acceptable, target-specific interventions.
Gupta et al., 2020 [23]	Prospective observational	Adult cancer patients from Karnataka	Inadequate health literacy and cognitive impairment were associated with severe adverse drug reactions to chemotherapy.
Harding, 2022 [24]	Qualitative	Cancer patients, families, and clinicians from Southern India	Families often conceal cancer diagnoses, leading to poor patient outcomes and a lack of agency in decision-making.
Jagan, 2018 [25]	Cross-sectional	School teachers from Karnataka	Conceptual oral health knowledge was fair; it was influenced by gender, age, education, and income and was associated with periodontal health.
Johri, 2015 [5]	Cross-sectional	Mothers in Uttar Pradesh and New Delhi	Maternal health literacy was associated with higher child vaccination completion rates in both rural and urban settings.
Johri, 2016 [4]	Cross-sectional	Mothers with children from Uttar Pradesh and New Delhi	High maternal health literacy was associated with severe child stunting and being severely underweight.
Khanna & Khanna, 2023 [26]	Letter to the Editor	General population	Highlights a significant research gap on the role of health literacy in India's extremely low cancer screening coverage.
Konsam et al., 2023 [27]	Randomised controlled trial	Primigravid women from South India	A combined health literacy and relaxing music intervention significantly improved sleep quality and reduced the risk of antenatal depression.
Mittal, 2023 [28]	Cross-sectional	Adult population from Haryana	Validated the All Aspects of Health Literacy Scale (AAHLS) in Hindi; found that higher socioeconomic status was associated with higher scores.
Muniyandi, 2015 [29]	Cross-sectional	Saharia tribal population from Madhya Pradesh	48% had never heard of tuberculosis (TB). Health literacy was very low, with general education being the only independent predictor.
Nagarjuna, 2023 [2]	Cross-sectional	Hypertensive patients from a Maharashtra urban slum	Most participants (76.9%) had low health literacy, which was significantly associated with poor self-care management of hypertension.
Ogorchukwu, 2016 [30]	Cross-sectional	Adolescents from Karnataka	Mental health literacy was very low; adolescents preferred informal help sources over formal ones due to pervasive stigma.
Passi et al., 2023 [8]	Cross-sectional	Residents of a resource-poor village in Chandigarh	Identified eight distinct health literacy profiles, with challenges in actively managing health and finding good health information.
Rathnakar U.P. et al., 2013 [6]	Cross-sectional	Patients in a tertiary care hospital in Karnataka	Younger age, higher education, and having a family physician were associated with better scores.
Saini, 2023 [31]	Experimental study	Soldiers in Pune and Panchkhula	A community-based psycho-educational module significantly improved mental health literacy among troops, with gains stable at six months.
Saraf, 2018 [32]	Cross-sectional	Adolescent girls in a Bengaluru urban slum	Mental health literacy regarding depression was low; stigma and lack of awareness were major barriers to seeking professional help.
Sharma et al., 2021 [33]	Intervention evaluation	University students from Haryana	A mobile health intervention was a cost-effective method for improving eye health literacy.
Shikha et al., 2023 [34]	Editorial	General	Emphasises that health literacy is crucial for maternal health, NCD prevention, and public health emergencies, influenced by diverse determinants.
Sil et al., 2017 [7]	Cross-sectional	Caregivers of sick children from Eastern India	While caregivers showed good practical skills, significant knowledge gaps existed regarding medicine dosing, storage, and dosage intervals.
Singh, 2018 [35]	Cross-sectional	Adult diabetic patients from Karnataka	63% of diabetic patients had low health literacy, which was significantly correlated with poorer understanding of prescription instructions.
Sowmya et al., 2021 [36]	Cross-sectional	Pre-school children and their mothers in Bangalore	Mothers' oral health literacy and behaviour were significantly associated with their children's dental caries experience.
Wu and Dubé, 2021 [37]	Cross-sectional	Rural households from Odisha	Higher home-grown food consumption was associated with higher fruit and vegetable intake, and this effect was concentrated among those with low health literacy.

Determinants of Health Literacy

These are the upstream, socio-ecological factors that shape an individual's health literacy. Education is consistently identified as one of the strongest predictors. A study among diabetic patients in Mangalore found a significant association between educational status and health literacy [35]. Similarly, a study in Gujarat identified illiteracy as a significant factor associated with a higher likelihood of tobacco consumption [3]. Socioeconomic status is also critical; inadequate oral health literacy was found to be significantly associated with lower and lower-middle socioeconomic status [2,19]. Age and gender were also significant determinants [5,6,25].

Systemic barriers compound individual factors. A profound urban-rural divide exists, with rural populations having significantly lower health literacy on diseases like tuberculosis [29]. Social structures, such as caste, were also found to be strongly associated with health literacy levels [8]. Finally, India's vast linguistic diversity [20] creates further barriers, necessitating tools adapted to local contexts and languages [21,28].

Core Dimensions of Health Literacy

This is the central, multidimensional capacity that is influenced by the determinants. The data highlights a set of core skills [38]. These include Functional Literacy (basic reading/numeracy) [7,35], Communicative/Interactive Literacy (engaging with providers), and Critical Literacy (appraising information) [28]. The literature maps to a four-step patient journey of accessing information and care, understanding it, appraising its quality, and applying it to make a decision [2,4,28].

Mediating Pathways

These are extra steps that operate between the central components of health literacy and poor health outcomes. Low literacy is linked to delayed care-seeking. This delay is often mediated by a fear of "loss of daily wages", weighed against the perceived health risk [8]. Poor health literacy is significantly associated with poor self-care practices for non-communicable diseases (NCDs) like hypertension [2]. A lack of knowledge (functional literacy) is a significant predictor of harmful behaviours, such as tobacco use [3]. Higher maternal health literacy is strongly associated with better child nutritional status, an outcome of preventive behaviours [4]. Low parental health literacy is associated with pediatric medication dosing errors [7].

Health and System-Level Outcomes

These pathways culminate in population-level outcomes. Low health literacy is strongly linked to poor self-care management of hypertension [2]. High maternal health literacy was found to be strongly protective, halving the likelihood of severe child stunting [4]. Gaps in mental health literacy compromise help-seeking for conditions like depression and antenatal depression [27,30]. A critical outcome is the level of trust in the health system; mistrust and communication barriers act as a barrier to care [24].

Key Feedback Loops

The three key feedback loops that governed the behaviour of this dynamic system (Figure 2) are listed below.

R1 - The health disparity trap (vicious cycle): This loop illustrates how socioeconomic disadvantage is translated into poor health, thereby reinforcing the initial disadvantage. Lower socioeconomic status and education [19,6] lead to low health literacy, which leads to poor health behaviours [3,7] and poor self-care [2]. This results in poor health outcomes (e.g., poor hypertension management; child stunting [2,4]), which leads to reduced economic productivity [8], reinforcing the lower socioeconomic status.

R2 - The system trust spiral (vicious cycle): This loop explains how the system resists improvement. Poor outcomes and difficult-to-navigate systems (driven by R1) lead to mistrust in the health system [24]. This mistrust fosters care avoidance and delayed care-seeking, allowing conditions to worsen and leading to even poorer health outcomes, which further reinforces the initial mistrust.

B1 - The community empowerment cycle (balancing loop): This loop represents the primary pathway for intervention. The data show that targeted interventions are effective [31,33]. They lead to improved health behaviours, such as increased birth preparedness, which in turn lead to better health outcomes, e.g., reduced antenatal depression [15,27]. This improvement "closes the gap," counteracting the negative loops.

Policy Scenarios Simulation Results

**Figure 3:**
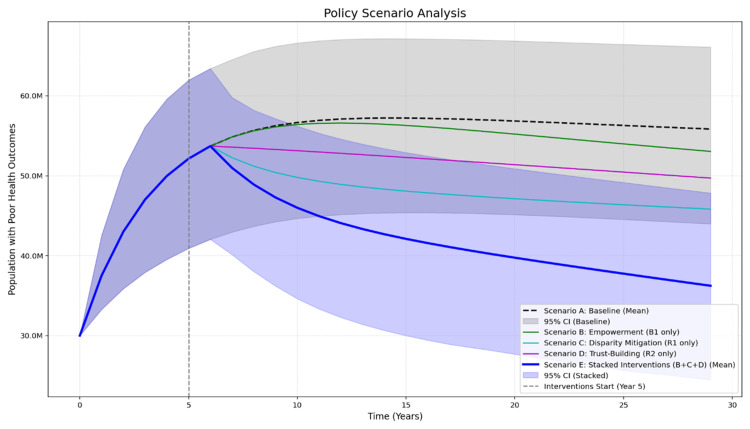
Policy Scenarios Analysis

We simulated five policy scenarios over 30 years to compare their effectiveness in reducing the population with poor health outcomes. The mean results of all five scenarios are presented in Figure 3.

The baseline (Scenario A), or do nothing, showed a steady increase in the population with poor health, stabilising at a mean of 55.6 million with 95% CI 43.8M-65.5M by Year 30. The 'Health Disparity Trap' (R1) is fully active, with low health literacy driving a high sickness rate. The 'System Trust Spiral' (R2) is also active; as the population with poor health outcomes remains high, trust continuously erodes, suppressing any natural improvement in health literacy. The 'Community Empowerment Cycle' (B1) is inactive, with only a small baseline rate of health literacy improvement occurring, leading to the system being "stuck" in its vicious cycles, causing the population with poor health outcomes stock to fill and stabilise at the worst possible outcome.

Scenario B (Empowerment) only reduced the mean population with poor health outcomes to 52.6M. This scenario activates the B1 loop by applying the effectiveness of the intervention [15,17]. The health literacy improvement flow is turned on, accelerating the movement of people from the population with low health literacy to the population with high health literacy stock. This intervention does not directly mitigate the high sickness rate from R1, and its effectiveness is actively suppressed by the policy resistance created by eroding trust from the R2 loop.

Scenario C (Disparity Mitigation) was the most effective single lever, reducing the mean population with poor health outcomes to 45.5 million. This scenario tests a hypothesised social support policy. While it is the most direct and effective single intervention, it treats the symptom (poor health) without addressing the root causes (low health literacy and low system trust).

Scenario D (Trust-Building) reduced the mean population with poor health outcomes to 49.4 million. This scenario tests a policy focused on improving healthcare quality and system trust. A hypothesised intervention, such as provider communication training, that addresses the R2 loop. This policy acts in two ways: it reduces the total sickness inflow to simulate higher quality care, and it activates the trust-building flow to begin refilling the public trust stock. It helps improve outcomes and system resilience, but fails to address the large population with low health literacy.

The Stacked Intervention (Scenario E), which combined all three policy levers simultaneously, was the only scenario to show a powerful, synergistic effect, reducing the mean population with poor health outcomes to 35.7 million. The interventions cease to conflict and begin to reinforce one another. The trust-building flow refills the public trust stock, which lifts the policy resistance and amplifies the power of the Empowerment intervention (Scenario B). This, in turn, accelerates the depletion of the high-risk population with low health literacy stock, weakening the R1 disparity trap. As the population's poor health outcomes stock falls due to the combined effects of Scenarios B, C, and D, the trust erosion flow of the R2 loop breaks, creating a virtuous cycle where rising trust further boosts empowerment. This is the only scenario designed to break both vicious cycles (R1 and R2) while strengthening the virtuous cycle (B1). However, its 95% confidence interval (CI: 24.9M-47.6M) still showed a minor overlap with the lower bound of the baseline's CI (43.8M).

Discussion

The model demonstrates that the system is currently dominated by two powerful, reinforcing (vicious) cycles: the "Health Disparity Trap" (R1) and the "System Trust Spiral" (R2). R1 shows how social and economic disadvantage are converted into poor health, which in turn reinforces that disadvantage. R2 explains why this inequity is so persistent, as it erodes the trust required for the system to function, leading to a cycle of disengagement.

Our simulation shows that a standalone empowerment program (Scenario B) is likely to underperform. Our model suggests this is due to "policy resistance" from the R2 loop; as trust erodes, the effectiveness of the empowerment intervention is suppressed. This simulated 'policy resistance' aligns with qualitative evidence from the review, which found that significant mistrust and challenging family dynamics in clinical settings can create barriers to patient agency and informed decision-making, even when relevant health information is available [24]. The Stacked Intervention (Scenario E) produced a mean result (35.7M) far superior to any isolated intervention, suggesting a powerful synergistic effect. However, the most critical finding from our sensitivity analysis is the persistent uncertainty. The overlap between the 95% CIs of the stacked (24.9M-47.6M) and baseline (43.8M-65.5M) scenarios indicates uncertainty in the effectiveness, given the wide variance in the parameters. Future research could potentially address this uncertainty and improve model precision through more precise effect size estimates.

## Conclusions

This is the first study in India to apply system dynamics modelling to synthesise the evidence on health literacy, providing a novel tool for understanding systemic feedback and policy simulation. It demonstrates that the system is dominated by reinforcing feedback loops that link social disadvantage, low literacy, and system mistrust, perpetuating health inequities. Our model provides a quantitative argument that to solve this complex problem, we must move from isolated projects to a sustained, systemic, and multi-pronged strategy. However, our simulation also highlights that the high variance in intervention effectiveness is a critical barrier, emphasising the need for more robust, scalable intervention models and research to validate them.

## Appendices

Appendix 1: PubMed search string

( "health literacy"[Title/Abstract] OR "health-literate"[Title/Abstract] OR "health literac*"[Title/Abstract] OR "functional health literacy"[Title/Abstract] OR "communicative health literacy"[Title/Abstract] OR "critical health literacy"[Title/Abstract] OR "Health Literacy"[Mesh] ) AND ( India[Title/Abstract] OR India[Affiliation] OR Indian[Title/Abstract] OR "Andhra Pradesh"[Title/Abstract] OR "Arunachal Pradesh"[Title/Abstract] OR "Assam"[Title/Abstract] OR "Bihar"[Title/Abstract] OR "Chhattisgarh"[Title/Abstract] OR "Goa"[Title/Abstract] OR "Gujarat"[Title/Abstract] OR "Haryana"[Title/Abstract] OR "Himachal Pradesh"[Title/Abstract] OR "Jharkhand"[Title/Abstract] OR "Karnataka"[Title/Abstract] OR "Kerala"[Title/Abstract] OR "Madhya Pradesh"[Title/Abstract] OR "Maharashtra"[Title/Abstract] OR "Manipur"[Title/Abstract] OR "Meghalaya"[Title/Abstract] OR "Mizoram"[Title/Abstract] OR "Nagaland"[Title/Abstract] OR "Odisha"[Title/Abstract] OR "Punjab"[Title/Abstract] OR "Rajasthan"[Title/Abstract] OR "Sikkim"[Title/Abstract] OR "Tamil Nadu"[Title/Abstract] OR "Telangana"[Title/Abstract] OR "Tripura"[Title/Abstract] OR "Uttar Pradesh"[Title/Abstract] OR "Uttarakhand"[Title/Abstract] OR "West Bengal"[Title/Abstract] ).

Appendix 2: Glossary of key terms [9]

System Dynamics Modelling (SDM): A methodological approach to understanding the nonlinear behaviour of complex systems over time using stocks, flows, internal feedback loops, and time delays.

Causal Loop Diagram (CLD): A visual representation of the dynamic interrelationships between variables in a system. It identifies circular chains of cause and effect (feedback loops) rather than linear relationships.

Stock: An accumulation within the system that characterises the state of the system at any point in time (e.g., the number of people with low health literacy). Stocks provide the system with memory and inertia.

Flow: The rate at which a stock changes over a specific period (e.g., the rate at which people learn new health skills per year). Flows are the only variables that can directly increase or decrease a stock.

Feedback Loop: A closed chain of causal connections.

Reinforcing Loop (R): A positive feedback structure that amplifies change, leading to exponential growth or collapse (e.g., a "vicious cycle").

Balancing Loop (B): A negative feedback structure that counteracts change, seeking to stabilize the system or move it toward a goal.

Policy Resistance: The tendency of a complex system to defeat or diminish the effects of an intervention because of the system's internal feedback structure (e.g., when mistrust lowers the uptake of a free health program).

Appendix 3: Model architecture

The simulation is a stock and flow model with four primary stocks. The model operates over a 30-year simulation period with a time step of 1 year.

Population with Low Health Literacy

Represents the segment of the population with inadequate health literacy, considered the "at-risk" group. This stock is depleted by the HL Improvement. A multiplier derived from Kulkarni et al. [2] is applied to this population, increasing their probability of entering the Poor Health stock by a factor of 3.5 compared to the baseline sickness rate.

Initial State: 80 million individuals (80% of total population).

Population with High Health Literacy

Represents the segment of the population with adequate or sufficient health literacy. This stock accumulates individuals transitioning from the Low HL stock. A protective multiplier derived from Johri et al. [4] is applied here, reducing the probability of entering the Poor Health stock by 50% (AOR 0.50) relative to the baseline.

Initial State: 20 million individuals (20% of total population)

Population with Poor Health Outcomes

The stock represents the aggregate adverse health outcomes of the population. If the prevalence of poor health exceeds a calibrated threshold of 30%, trust erosion is triggered. Inflow into this stock is determined by the sum of sickness incidence of population with low and high Health Literacy, and the outflow is determined by the Recovery Flow.

Initial State: 30 million individuals (30% prevalence).

Public Trust

A dimensionless index scaled from 0 to 100, representing the population's confidence in the healthcare system. The efficiency of health literacy interventions is multiplied by a trust factor. To simulate policy resistance , as trust declines, the conversion of individuals from Low to High HL becomes less efficient.

Initial State: 70/100.

Dynamics:

Inflows: Trust Building Flow (activated only by specific policy interventions).

Outflows: Trust Erosion Flow (activated endogenously when health outcomes degrade).

Appendix 4: Model parameters

Parameter values, distributions used for sensitivity analysis, and source attributions are detailed in Table 2. For the sensitivity analysis, a Monte Carlo simulation (N=100 runs) was conducted. Data-driven parameters were sampled from Normal distributions derived from reported 95% Confidence Intervals (CIs). Assumed parameters were subjected to variation of +/- 20 percentage to test robustness.

**Table 2:** Model Parameters and Data Sources

Parameter Description	Data type	Base Value / Distribution	Source / Rationale
Low HL Risk Multiplier	Data Driven	AOR 3.5 (CI: 1.8–6.9)	Gautam et al., 2021. [16].
High HL Protective Multiplier	Data Driven	AOR 0.50 (CI: 0.33–0.74)	Johri et al., 2016. [4].
Intervention Efficacy	Data Driven	AOR 2.14 (CI: 1.73–2.65)	Ahmad et al., 2021. [17].
Initial Poor Health	Assumed	30,000,000 (30%)	Represents baseline disease burden.
Base Sickness Rate	Assumed	0.05 (Range: 0.04–0.06)	Annual incidence rate of poor health (5%) in absence of HL modifiers.
Base Recovery Rate	Assumed	0.10 (Range: 0.08–0.12)	Annual natural recovery rate (10%).
Base HL Improvement	Assumed	0.01 (Range: 0.008–0.012)	Background rate of HL gain (1% annually) via general education.
Social Support Impact	Assumed	1.5 (± 0.1)	Scenario C assumption: 50% increase in recovery rate.
Provider Training Impact	Assumed	0.8 (± 0.05)	Scenario D assumption: 20% reduction in sickness inflow.
Trust Erosion Sensitivity	Assumed	0.1 (± 0.03)	Coupling coefficient linking Poor Health prevalence to Trust loss.

Appendix 5: Policy scenario protocols

Interventions are introduced at Year 5.

Scenario A: Baseline

To simulate the natural trajectory of the system without external intervention. The Disparity Trap (R1) and Trust Spiral (R2) loops remain active and unchecked. The Empowerment Cycle (B1) remains at nominal baseline levels. Serves as the counterfactual reference for comparative analysis.

Scenario B: Community Empowerment

To test the efficacy of a standalone health literacy educational intervention. The flow rate from low health literacy stock to the high health literacy stock is multiplied by the Intervention Effect parameter (AOR ~2.16). This scenario does not mitigate the baseline sickness rate, nor does it exogenously repair public trust.

Scenario C: Disparity Mitigation

To test a social protection policy that addresses health outcomes directly (e.g., free treatment, nutritional support). The Recovery Flow leaving the Population with Poor Health Outcomes Stock is increased by 50%. This serves as a symptomatic treatment; it reduces the stock of poor health but does not alter the upstream distribution of health literacy.

Scenario D: Trust-Building

To test structural reforms in healthcare delivery (e.g., provider communication training). Reduces the Base Sickness Rate by 20% (simulating improved care quality). Activates an exogenous Trust Building Flow to replenish the Trust stock.

Scenario E: Stacked Strategy

To test the combined effect of multi-modal interventions. Simultaneously activates Scenarios B, C, and D. The replenishment of Trust (from D) enhances the efficiency of the Empowerment intervention (from B). Simultaneously, the direct reduction of Poor Health (from C) deactivates the Trust Erosion feedback loop.

## References

[1] Sørensen K, Van den Broucke S, Fullam J, Doyle G, Pelikan J, Slonska Z, Brand H (2012). Health literacy and public health: a systematic review and integration of definitions and models. BMC Public Health.

[2] Nagarjuna P, Kumar V, Faujdar DS, Yadav AK (2023). Role of health literacy and primary health-care access in self-care management of hypertension. Indian J Public Health.

[3] Chauhan D, Patel D, Yogesh M, Trivedi N (2024). Health literacy and tobacco cessation among hypertensive individuals: a mixed method study. J Educ Health Promot.

[4] Johri M, Subramanian SV, Koné GK (2016). Maternal health literacy is associated with early childhood nutritional status in India. J Nutr.

[5] Johri M, Subramanian SV, Sylvestre MP (2015). Association between maternal health literacy and child vaccination in India: a cross-sectional study. J Epidemiol Community Health.

[6] Rathnakar UP, Belman M, Kamath A, Unnikrishnan B, Shenoy AK, Udupa AL (2013). Evaluation of health literacy status among patients in a tertiary care hospital in coastal Karnataka, India. J Clin Diagn Res.

[7] Sil A, Sengupta C, Das AK, Sil PD, Datta S, Hazra A (2017). A study of knowledge, attitude and practice regarding administration of pediatric dosage forms and allied health literacy of caregivers for children. J Family Med Prim Care.

[8] Passi R, Kaur M, Lakshmi PV, Cheng C, Hawkins M, Osborne RH (2023). Health literacy strengths and challenges among residents of a resource-poor village in rural India: epidemiological and cluster analyses. PLOS Glob Public Health.

[9] Sterman JD (2000). Business Dynamics: Systems Thinking and Modeling for a Complex World. Boston, MA: Irwin/McGraw-Hill.

[10] Homer JB, Hirsch GB (2006). System dynamics modeling for public health: background and opportunities. Am J Public Health.

[11] Page MJ, McKenzie JE, Bossuyt PM (2021). The PRISMA 2020 statement: an updated guideline for reporting systematic reviews. BMJ.

[12] Van Rossum G, Drake FL (2009). Python 3 Reference Manual.

[13] Harris CR, Millman KJ, van der Walt SJ (2020). Array programming with NumPy. Nature.

[14] Hunter JD (2007). Matplotlib: a 2D graphics environment. Comput Sci Eng.

[15] Ahmad D, Mohanty I, Niyonsenga T (2022). Improving birth preparedness and complication readiness in rural India through an integrated microfinance and health literacy programme: evidence from a quasi-experimental study. BMJ Open.

[16] Gautam V, S D, Rustagi N (2021). Health literacy, preventive COVID 19 behaviour and adherence to chronic disease treatment during lockdown among patients registered at primary health facility in urban Jodhpur, Rajasthan. Diabetes Metab Syndr.

[17] Ahmad D, Mohanty I, Hazra A, Niyonsenga T (2021). The knowledge of danger signs of obstetric complications among women in rural India: evaluating an integrated microfinance and health literacy program. BMC Pregnancy Childbirth.

[18] Baliga SM (2019). Child oral health-care literacy in India: can access to services be improved?. J Indian Soc Pedod Prev Dent.

[19] Das D, Menon I, Gupta R, Arora V, Ashraf A, Ahsan I (2020). Oral health literacy: a practical strategy towards better oral health status among adult population of Ghaziabad district. J Family Med Prim Care.

[20] Douglass K, Narayan L, Allen R, Pandya J, Talib Z (2021). Language diversity and challenges to communication in Indian emergency departments. Int J Emerg Med.

[21] Dsouza JP, Van den Broucke S, Pattanshetty S (2021). Validity and reliability of the Indian version of the HLS-EU-Q16 questionnaire. Int J Environ Res Public Health.

[22] Gokdemir O, Kushwaha P, Shikha D, Petrazzuoli F, Bhattacharya S (2024). Editorial: Health literacy and disease prevention, volume II. Front Public Health.

[23] Gupta V, Shivaprakash G, Bhattacherjee D, Udupa K, Poojar B, Sori R, Mishra S (2020). Association of health literacy and cognition levels with severity of adverse drug reactions in cancer patients: a South Asian experience. Int J Clin Pharm.

[24] Harding R, Salins N, Sharan K, Ekstrand ML (2022). Health literacy in communication, decision-making and outcomes among cancer patients, their families and clinicians in India: a multicentre cross-sectional qualitative study. Psychooncology.

[25] Jagan P, Fareed N, Battur H, Khanagar S, Manohar B (2018). Conceptual knowledge of oral health among school teachers in South India, India. Eur J Dent.

[26] Khanna D, Khanna AK (2023). Research gap in health literacy: are we overlooking a possible solution to inadequate cancer screening in India?. Asian Pac J Cancer Prev.

[27] Konsam M, Praharaj SK, Panda S, Shetty J, Ravishankar N, D'Souza SR (2023). Effectiveness of health literacy and relaxing music on quality of sleep and risk for antenatal depression. Indian J Psychiatry.

[28] Mittal N, Nehra D, Mittal R, Gupta T (2023). Linguistic adaptation and validation of All Aspects of Health Literacy Scale (AAHLS): a health literacy assessment tool for use in Hindi-speaking population. Natl Med J India.

[29] Muniyandi M, Rao VG, Bhat J, Yadav R, Sharma RK, Bhondeley MK (2015). Health literacy on tuberculosis amongst vulnerable segment of population: special reference to Saharia tribe in central India. Indian J Med Res.

[30] Ogorchukwu JM, Sekaran VC, Nair S, Ashok L (2016). Mental health literacy among late adolescents in South India: what they know and what attitudes drive them. Indian J Psychol Med.

[31] Saini R, Das RC, Chatterjee K, Srivastava K, Khera A, Agrawal S (2023). Augmenting mental health literacy of troops in a large military station: a novel approach. Ind Psychiatry J.

[32] Saraf G, Chandra PS, Desai G, Rao GN (2018). What adolescent girls know about mental health: findings from a mental health literacy survey from an urban slum setting in India. Indian J Psychol Med.

[33] Sharma IP, Chaudhry M, Sharma D, Kaiti R (2021). Mobile health intervention for promotion of eye health literacy. PLOS Glob Public Health.

[34] Shikha D, Kushwaha P, Gokdemir O, Marzo RR, Bhattacharya S (2023). Editorial: Health literacy and disease prevention. Front Public Health.

[35] Singh S, Acharya SD, Kamath A, Ullal SD, Urval RP (2018). Health literacy status and understanding of the prescription instructions in diabetic patients. J Diabetes Res.

[36] Sowmya KR, Puranik MP, Aparna KS (2021). Association between mother's behaviour, oral health literacy and children's oral health outcomes: a cross-sectional study. Indian J Dent Res.

[37] Wu YH, Moore S, McRae C, Dubé L (2021). Tracing the single and combined contributions of home-grown supply and health literacy on fruit and vegetable consumption: an empirical exploration in rural India. Front Public Health.

[38] Nutbeam D (2000). Health literacy as a public health goal: a challenge for contemporary health education and communication strategies into the 21st century. Health Promot Int.

